# Psychosocial factors account for a proportion of the difference in cognitive performance between persons with and without HIV

**DOI:** 10.1097/QAD.0000000000004080

**Published:** 2024-12-19

**Authors:** Anna Jane Dreyer, Celine Le Roux, Kevin G.F. Thomas, Caroline A. Sabin, Alan Winston, Saye Khoo, John A. Joska, Sam Nightingale

**Affiliations:** a Department of Clinical Neurosciences, University of Cambridge, Cambridge, UK; b HIV Mental Health Research Unit, Division of Neuropsychiatry, Department of Psychiatry and Mental Health, Neuroscience Institute, University of Cape Town, Cape Town; c Faculty of Humanities, University of Pretoria, Pretoria, South Africa; d Institute for Global Health, University College London; e Imperial College Healthcare NHS Trust; f Department of Infectious Disease, Imperial College London, London; g Department of Pharmacology and Therapeutics. University of Liverpool, Liverpool, UK.

**Keywords:** cognitive assessment, cognitive impairment, HIV, HIV-associated neurocognitive disorders, psychosocial factors

## Abstract

**Objective:**

To investigate whether psychosocial factors account for a proportion of the difference in cognitive performance between persons with and without HIV.

**Design:**

Cross-sectional study of 273 participants (178 persons with HIV) from a low income area of Cape Town, South Africa,

**Methods:**

Participants completed comprehensive cognitive testing (7 domains) and 12 psychosocial measures (5 current: income, occupation, assets, accommodation, depressive symptoms, 7 from childhood: assets, quality of education, exposure to childhood trauma and violence, primary caregiver occupation and highest level of education), as well as demographic measures standard in cognition studies (age, sex, years of education). We investigated the HIV association with global cognitive performance after adjustment for standard demographic variables, exploratory psychosocial variables, and balancing characteristics of those with and without HIV using propensity score modelling.

**Results:**

Persons with HIV had significantly lower scores than persons without HIV in 8/12 psychosocial variables. Of these, 7/12 significantly predicted global T-score. In unadjusted regression, HIV status was associated with a reduction in global T-score of 3.72 units. Adjustment for standard variables, reduced the effect of HIV on global T score by 26.9% to 2.72, additional adjustment for psychosocial variables reduced by 40.3% to 2.22, and adjustment for propensity scores by 42.7% to 2.13.

**Conclusions:**

Persons with HIV in this setting have lower psychosocial indices, both current and in childhood, which are associated with lower cognitive test performance as an adult. This is incompletely mitigated by adjustments for standard demographic variables which risks overestimation of cognitive impairment on a population level.

## Introduction

It has been widely reported that cognitive impairment remains common in persons with HIV; a 2021 meta-analysis of 123 studies including 35 513 participants estimated that 43% of persons with HIV globally had HIV-associated neurocognitive disorders (HAND), with higher rates estimated in Africa [1].

The prevalence of cognitive impairment in persons with HIV is however, the subject of controversy [2]. Accurate assessment of cognitive performance requires consideration of a range of complex psychosocial factors, including social stressors, poverty and limited educational attainment as well as by disorders that induce brain injury [3–5], many of which disproportionately affect persons with HIV [6,7]. As such, there is the possibility that low cognitive performance seen in persons with HIV could be falsely attributed to the direct effects of HIV on the brain, rather than these psychosocial factors. This over attribution to HIV-associated brain injury could lead to misguided investigation in biomarker and treatment research. The 2007 HAND criteria [8] have been criticized for overreliance on psychometric neuropsychological methods and estimates using this criterion far exceed rates seen in clinical practice [9–12]. Overestimation of the prevalence of cognitive impairment in persons with HIV could compound stigma and discrimination towards this already marginalized group.

Within research studies, the approach taken to controlling for the confounding that might be introduced by these psychosocial factors typically includes the use of matched normative data and adjustment for standard demographic variables (age, sex, ethnicity and years of education) [13]. However, in multicultural and multilingual countries like South Africa that also have significant population-wide psychosocial and socioeconomic disparities, it is difficult to obtain well matched normative data [14]. Moreover, a group of persons with HIV cannot be assumed to have comparable psychosocial and socioeconomic opportunities to persons without HIV, given the complex psychosocial factors associated with HIV acquisition risk even if both groups are from apparently similar contexts [7,15]. The term ‘psychosocial factors’ covers a range of psychological characteristics and social experiences, including socioeconomic status. Childhood psychosocial factors may exert a greater influence on outcomes as an adult than adult circumstances, but are rarely measured in research studies. In this study, we were interested in whether current psychosocial circumstances (income, occupation, assets, accommodation, depressive symptoms), and those experienced in childhood (assets, quality of education, exposure to childhood trauma and violence, primary caregiver occupation and level of education) were associated with performance on cognitive tests. These were selected to reflect the psychosocial background of the population in this area with some shown in previous studies to be associated with cognitive performance [3,16].

We hypothesized that psychosocial disadvantage amongst persons with HIV in low-income areas of Cape Town, South Africa, is associated with lower cognitive performance. Furthermore, that the bias introduced by this in analyses comparing cognitive performance in persons with and without HIV is incompletely mitigated by correction for standard demographic variables typically controlled for in HIV-cognition studies, that is, age, sex, ethnicity and years of education.

## Methods

### Participants and setting

Participants were recruited as part of the CONNECT (COgnition, Neuropsychiatric Symptoms and NEuroinflammation Switching to Dolutegravir in Cape Town) study. Study details described previously [17].

Persons with HIV were recruited from the HIV clinic in Gugulethu, a peri-urban area of Cape Town, South Africa, where majority of residents are isiXhosa-speaking Black Africans, and there are high levels of poverty [18].

Eligible persons with HIV were adults (18–55 years) who had been receiving efavirenz-based antiretroviral therapy for at least 1 year who had been identified by the clinic as suitable candidates for the switch to dolutegravir-based antiretroviral therapy as per the South African national HIV treatment guidelines (a measured HIV RNA <50 copies/ml on a single sample, or <1000 copies/ml on two samples taken 3 months apart) [19].

Persons without HIV were also recruited from Gugulethu and were matched to persons with HIV by sex and age (within 5 years). To recruit persons without HIV with as similar socioeconomic indices as possible to those with HIV, we recruited friends, relatives, and associates of persons attending the Gugulethu HIV clinic. HIV status was confirmed to be negative in this group by rapid test.

We excluded from participation all individuals with current substance misuse (drug use disorders identification test >5 for men or >1 for women); high-risk or harmful level alcohol use (alcohol use disorders identification test >15) [20]; history of central nervous system (CNS) infection or major head injury (loss of conscious >30 min); uncontrolled neurologic conditions (e.g., seizure disorders, established cerebrovascular disease); history of learning difficulty or severe intellectual disability; <7 years total education; history of severe mental health disorder (schizophrenia, psychosis or bipolar disorder); or vertical HIV acquisition for those with HIV. We also excluded individuals who at the time of enrolment were being investigated or treated for active intercurrent illness such as infection or carcinoma, were currently receiving treatment for tuberculosis, were known or suspected to be pregnant, or were not fluent in isiXhosa or English.

All measures were translated into isiXhosa and administered and scored by a trained isiXhosa speaking technician in the participants’ language of choice. A registered clinical neuropsychologist (AJD) supervised test administration and scoring protocols. The study was approved by University of Cape Town Faculty of Health Sciences Human Research Ethics Committee (017/2019).

## Measures

### Cognitive tests and outcomes

Neuropsychological test performance was evaluated using a standard battery taking approximately 2 h assessing the cognitive domains of executive functioning, verbal learning and memory, visuospatial learning and memory, verbal fluency, attention/working memory, information processing speed, and motor skills [21,22].

Raw scores for all participants were standardized to *z*-scores using the mean and standard deviation in those without HIV (*N* = 95). The *z*-scores were then converted to T-scores [mean = 50, standard deviation (SD) = 10]. Domain T-scores were calculated by taking the average of T-scores of the cognitive outcomes within each domain. The global T-score was calculated by taking the average across domain T-scores.

Further details of cognitive tests and outcomes, and the processing of cognitive data into *z*-scores and T-scores, are provided in supplementary materials.

### Standard demographic variables

We collected data on parameters typically controlled for in HIV-cognition studies and specified in HAND criteria: that is, age, sex, ethnicity and years of formal education [13].

### Psychosocial indices

We applied measures of 12 psychosocial variables, 5 which reflect current status and 7 recalled at childhood.

#### Current psychosocial variables

*Monthly income* from all sources before tax was collected in nine brackets spanning ZAR0 to >ZAR20 000. *Occupation category* was based on Hollingshead occupational scale (see Table 1 for list) [23]. The *Current asset index* was measured by asking the participant to record whether they had various items in working order in their household (see Table 1 for list); a point was given for each asset, with a possible total score of 14. *Accommodation type* was measured by asking the participant what type of housing they lived in: none, ‘shack’ (a small informal house constructed from corrugated iron sheets attached to a basic wooden frame), ‘wendyhouse or backyard dwelling’ (a wendyhouse is a small timber cabin), or ‘own or family house’ (usually a brick house). *Depressive symptomatology* was measured by the Center for Epidemiological Studies-Depression (CES-D) score with a standard cut off of ≥16 used to indicate depressive symptoms [24].

**Table 1:** Demographic and psychosocial characteristics of persons with and without HIV (*N* = 273).

		Persons with HIV (*N* = 178)		Persons without HIV (*N* = 95)	
Variables	*n*	Mean (SD) or *n* (%) as appropriate	*n*	Mean (SD) or *n* (%) as appropriate	*P*
**Standard demographic**					
Sex (male)	178	36 (20.2%)	95	23 (24.21%)	0.543
Age (years)	178	40.6 (7.5)	95	39.5 (9.16)	0.277
Years of education	178	10.7 (1.3)	94	11.2 (1.21)	<0.001
First language isiXhosa	178	164 (92.1%)	95	87 (91.6%)	0.109
**Current psychosocial**					
Occupation category	178		90		0.454^a^
Higher executives, major professionals, owners of large businesses		0 (0%)		0 (0%)	
Business managers of medium sized businesses, lesser professions		2 (1.1%)		0 (0%)	
Administrative personnel, managers, minor professionals, owners of small businesses		4 (2.3%)		0 (0%)	
Clerical and sales, technicians, small businesses		9 (5.1%)		7 (7.8%)	
Skilled manual		12 (6.7%)		3 (3.3%)	
Semi-skilled		29 (16.3%)		12 (13.3%)	
Unskilled		31 (17.4%)		14 (15.6%)	
Homemaker		8 (4.5%)		2 (2.2%)	
Student, disabled, no occupation		83 (46.8)		52 (57.8%)	
Monthly income from all sources (ZAR)	178		91		0.575
R0−R499		29 (16.3%)		13 (14.3%)	
R500−R999		18 (10.1%)		13 (14.3%)	
R1000−R1999		41 (23.0%)		21 (23.1%)	
R2000−R2999		18 (10.1%)		13 (14.3%)	
R3000−R3999		20 (11.2%)		11 (12.1%)	
R4000−R4999		22 (12.4%)		5 (5.5%)	
R5000−R9999		27 (15.2%)		12 (13.2%)	
R10 000−R19 000		3 (1.7%)		3 (3.3%)	
>R20 000		0 (0%)		0 (0%)	
Depressive symptoms (CES-D ≥16)	178	17 (9.6%)	95	21 (22.1%)	0.007
Accommodation type	178		91		<0.001
None		0 (0%)		0 (0%)	
Shack		66 (37.1%)		11 (12.1%)	
Wendyhouse or backyard dwelling		53 (29.8%)		18 (19.8%)	
Own or family house		59 (33.2%)		62 (68.3%)	
Current asset index	178		91		
Refrigerator or freezer		157 (88.2%)		85 (93.4%)	
Vacuum cleaner or polisher		10 (11.0%)		18 (10.1%)	
Television		157 (88.2%)		87 (95.6%)	
Hi-fi or music centre (radio excluded)		39 (42.9%)		60 (33.7%)	
Microwave oven		67 (73.6%)		121 (68.1%)	
Washing machine		68 (38.2%)		42 (46.2%)	
Video cassette recorder or DVD player		44 (48.4%)		78 (43.8%)	
Running water		151 (84.8%)		86 (94.5%)	
Domestic worker		3 (1.7%)		0 (0%)	
At least one car		27 (15.2%)		16 (17.6%)	
Flush toilet		115 (64.6%)		77 (84.6%)	
Built-in kitchen sink		72 (40.4%)		66 (72.5%)	
An electric stove or hotplate		169 (94.9%)		88 (96.7%)	
Working telephone		174 (97.8%)		86 (94.5%)	
Current asset index total score		7.7 (2.3)		8.7 (2.0)	<0.001
**Childhood psychosocial**					
Primary school quality of education quintile	141		81		0.003^a^
Quintile 1		27 (19.1%)		9 (11.1%)	
Quintile 2		31 (22.0%)		18 (22.2%)	
Quintile 3		79 (56.0%)		40 (49.4%)	
Quintile 4		3 (2.1%)		11 (13.6%)	
Quintile 5		1 (0.7%)		3 (3.7%)	
Secondary school quality of education quintile	148		80		0.012
Quintile 1		11 (7.4%)		6 (7.5%)	
Quintile 2		42 (28.4%)		13 (16.2%)	
Quintile 3		73 (49.3%)		37 (46.2%)	
Quintile 4		16 (10.8%)		11 (13.8%)	
Quintile 5		6 (4.1%)		13 (16.2%)	
Primary caregiver highest level of education	173		78		0.005^a^
0 years		24 (13.9%)		3 (3.9%)	
1–6 years		40 (23.1%)		17 (21.8%)	
7 years		24 (13.9%)		5 (6.4%)	
8–11 years		71 (41.0%)		36 (46.2%)	
12 years		9 (5.2%)		9 (11.5%)	
>13 years		5 (2.9%)		8 (10.3%)	
Primary caregiver occupation category	178		91		0.017
Higher executives, major professionals, owners of large businesses		0 (0%)		0 (0%)	
Business managers of medium sized businesses, lesser professions		6 (3.4%)		8 (8.8%)	
Administrative personnel, managers, minor professionals, owners of small businesses		2 (1.1%)		2 (2.2%)	
Clerical and sales, technicians, small businesses		4 (2.3%)		7 (7.7%)	
Skilled manual		7 (3.9%)		4 (4.4%)	
Semi-skilled		28 (15.7%)		19 (20.9%)	
Unskilled		39 (21.9%)		24 (26.4%)	
Homemaker		51 (28.7%)		18 (19.8%)	
Student, disabled, no occupation		41 (23.0%)		9 (9.9%)	
Childhood asset index					
Refrigerator or freezer		73 (41%)		53 (58.9%)	
Vacuum cleaner or polisher		11 (6.2%)		14 (15.6%)	
Television		82 (46.1%)		41 (62.2%)	
Hi-fi or music center (radio excluded)		67 (37.6%)		54 (60%)	
Microwave oven		33 (18.5%)		17 (18.9%)	
Washing machine		16 (9%)		15 (16.7%)	
Video cassette recorder or dvd player		40 (44.4%)		45 (25.3%)	
Running water		86 (48.3%)		68 (75.6%)	
Domestic worker		17 (9.6%)		10 (11.1%)	
At least one car		25 (14%)		25 (28.1%)	
Flush toilet		74 (41.6%)		60 (33.7%)	
Built-in kitchen sink		60 (33.7%)		62 (68.9%)	
An electric stove or hotplate		73 (41%)		52 (57.8%)	
Working telephone		72 (40.4%		50 (55.6%)	
Childhood asset index total score	177	4.1 (4.1)	89	6.5 (4.1)	<0.001
Childhood trauma (CTQ score)	178	36.5 (12.8)	91	35.1 (11.8)	0.120
Exposure to violence (SECTV score)	178	10.4 (7.9)	91	13.7 (7.8)	<0.001

CES-D, Centre for Epidemiological Studies-Depression; CTQ, Childhood Trauma Questionnaire; SECTV, Survey of Exposure to Community Violence; ZAR, South African Rands.

a Fisher's Exact Test instead of chi-square.

#### Childhood psychosocial variables

Participants were asked to recall the environmental circumstances to which they were exposed in childhood (aged 8–10 years). *Primary and secondary school quality of education* was measured by recording the name and province of the primary school (Grades 0–7, 6–13 years of age) and secondary school (Grades 8–12, 13–18 years of age) attended (if the participant reported attending more than one primary or secondary school, data were recorded for the school at which the longest time had been spent). Using this information, we classified the schools using the South African government's national quintile system 2020 rankings, rating from quintile 1 (poorest) to 5 (least poor). *Primary caregiver highest level of education* was recorded in six categories, ranging from ‘no formal education’ to ‘tertiary education’. The primary caregiver was defined as the biological mother if present; if not, then the father, and then a guardian. *Primary caregiver occupation category* was collected based on the Hollingshead occupational scale, as earlier [23]. *Childhood asset index* were measured using the same asset categories as earlier. *Childhood trauma* was measured using the Childhood Trauma Questionnaire (CTQ) recalled up to the age of 16 [25]. *Exposure to violence* was measured using the Survey of Exposure to Community Violence (SECTV). This questionnaire asks respondents to indicate how often they saw or heard violent things in their home, neighbourhood, or school (not on TV or in movies) when younger than 18 years of age.

### Statistical analysis

R version 4.3.1 (2023-06-16 ucrt) and RStudio version 2024.04.2 were used to complete all analyses, with the threshold for statistical significance set at *α* = 0.05.

We calculated descriptive statistics for all measures and assessed the normality of the distributions of continuous variables using the Shapiro–Wilk test. We then used *t*-tests (or Wilcoxon Rank Sum tests if statistical assumption of normality was not met) and chi-square analyses with Yates’ continuity correction (or Fisher's exact tests if any of the frequencies in each cell of the contingency table were <5) to investigate differences between the groups of persons with and without HIV.

We then conducted analyses to determine the contribution of the psychosocial factors to any differences in cognitive performance seen between those with and without HIV. We conducted univariable linear regression models to investigate the associations between each predictor variable and global cognitive performance (as measured by the global T-score). For these analyses, we used *income, occupational category, quality of education quintile and primary caregiver highest level of education* as numerical variables, with estimates relating to the association with each 1 point increment in global T-score.

Next, we used principal component analysis (PCA) on the continuous psychosocial variables to identify underlying patterns and reduce the dimensionality of the dataset for subsequent multivariable modelling. The PCA was conducted on the following variables: *CTQ score, SECTV score, primary caregiver occupation category, childhood asset index, primary school quality of education quintile, secondary school quality of education quintile, primary caregiver level of education, current asset index, monthly income* and *occupation category*. Cattell's criteria (i.e., examination of the scree plot) was used to determine the number of components to retain. We then fitted univariable linear regression models to describe the associations of the principal components with the global T-score.

Next, we then built two multivariable regression models, one adjusting for standard demographic variables only (i.e., *age, sex, years of education*), and the other with the additional adjustment of psychosocial variables (principal components, as well as *accommodation type and depressive symptoms)* to see whether the inclusion of the psychosocial variables attenuated the observed HIV association.

Finally, we used propensity score modelling to balance the characteristics of those with and without HIV and thus remove any potential confounding effects. We built the logistic regression models with HIV status as the outcome, and predictors being *age at consent, years of education, sex,* the principal components*, accommodation type, and depressive symptoms.* Based on the parameter estimates from this model, we generated propensity scores for each participant which gives an indication of the probability of an individual being in the HIV group given their observed characteristics. Analyses then included this propensity score as a covariate in addition to HIV status.

## Results

### Sample characteristics

Participants (*N* = 273) were recruited between August 2019 and September 2022: 178 persons with HIV and 95 persons without HIV. Most persons with HIV were virally suppressed with 92% having a plasma HIV RNA below 50 copies/ml, and 98% below 1000 [17]. Whilst the age and sex distributions were similar between the two groups, as was the proportion with a first language of isiXhosa, those with HIV had a significantly lower level of education than those without HIV (Table 1). Persons with HIV had significantly lower scores than persons without HIV on 8 of the 12 measured psychosocial variables (*P*-values < 0.017 for each).

### Univariable associations of standard demographic, current psychosocial, and childhood psychosocial variables with cognitive test performance

The mean (SD) global T-score was 47.60 (6.18) overall; 46.31 (6.01) in the group with HIV and 50.80 (5.80) in the group without HIV (mean difference −3.72 [95% confidence interval −5.21, −2.22], *P* = 0.001). The mean global T-score also declined by 0.24 per year increment in age, and increased by 2.05 for each additional year of education in the full group (Table 2).

**Table 2:** Univariable associations with global T-score.

Variables	Beta estimate	95% CI	*P*
**Standard demographic**			
HIV status (positive)	−3.72	−5.21 to −2.22	<0.001
Age (years)	−0.24	−0.32 to −0.15	<0.001
Sex (male)	−0.08	−1.88–1.71	0.929
Years of education	2.05	1.52–2.58	<0.001
**Current psychosocial**			
Monthly income from all sources (ZAR)	−0.03	−0.39–0.33	0.877
Occupation category	−0.16	−0.58–0.26	0.464
Accommodation type			
Wendyhouse/backyard dwelling vs. shack	3.41	1.45–5.37	0.001
Own/family house vs. shack	3.43	1.70–5.15	<0.001
Current asset index (per 1 point higher)	0.45	0.13–0.78	0.007
Depressive symptoms (CES-D ≥ 16)	1.86	−0.26–3.98	0.086
**Childhood psychosocial**			
Childhood asset index (per 1 point higher)	0.35	0.18–0.52	<0.001
Primary school quality of education (per quintile higher)	1.41	0.52–2.30	0.002
Secondary school quality of education (per quintile higher)	0.53	−0.25–1.31	0.180
Primary caregiver highest level of education (per year)	1.06	0.48–1.64	<0.001
Primary caregiver occupation category (per 1 point higher)	−0.58	−0.99 to −0.16	0.006
Childhood trauma (CTQ score)	−0.02	−0.08–0.04	0.601
Exposure to violence (SECTV score)	0.13	0.04–0.23	0.004

95% CI, 95% confidence interval; CES-D, Center for Epidemiological Studies-Depression; CTQ, Childhood Trauma Questionnaire; SECTV, Survey of Exposure to Community Violence; ZAR, South African Rands.

In total, 7 of the 12 measured psychosocial variables were significantly associated with global T-score (Table 2). People living in their own/family house or in a wendyhouse/backyard dwelling had better global cognitive performance compared to people living in a shack. Those with fewer assets as adults and as children had lower global cognitive performance. Attending a better quality of primary school, having a primary caregiver with higher level of education, and having a primary caregiver with higher occupation status, were each also associated with better global cognitive performance. Exposure to violence as a child was associated with better global cognitive performance.

The PCA revealed three components, labelled as *Childhood Psychosocial Variables*, *Current Psychosocial Variables* and *Experience of Childhood Trauma.* Details of the PCA results are in supplementary material (Table 1, Supplemental Digital Content). In univariate linear regression analyses of these three components, only the *Childhood Psychosocial Variables* was significantly associated with the global T-score (Table 3).

**Table 3:** Univariable associations of PCA components with global T-score.

Variables	Beta estimate	95% CI	*P*
*Childhood Psychosocial Variables* component	1.78	1.07–2.49	<0.001
*Current Psychosocial Variables* component	0.51	−0.23–1.25	0.174
*Experience of Childhood Trauma* component	−0.25	−0.99–0.49	0.505

95% CI, 95% confidence interval.

### HIV association with cognitive test performance after adjustment in multivariable models

Unadjusted, HIV status was associated with a reduction in global T-score of 3.72 units. After adjustment for the standard demographic variables alone, the HIV association reduced by 26.9% to 2.72. After additional adjustment for psychosocial variables, the HIV association reduced by 40.3% to 2.22. After adjustment for propensity score, the HIV association reduced by 42.7% to 2.13 (Table 4). The logistic regression model used to determine propensity score is reported in supplementary material (Table 2, Supplemental Digital Content).

**Table 4:** Association of HIV with global T score after various adjustments.

	Model 1: Unadjusted		Model 2: Adjustment for demographic variables only		Model 3: Adjustment for demographic factors and psychosocial variables (PCA)		Model 4: Adjustment for propensity score	
Variables	β (95% CI)	*P*	β (95% CI)	*P*	β (95% CI)	*P*	β (95% CI)	*P*
HIV status	−3.72 (−5.21, −2.22)	<0.001	−2.72 (−4.09, −1.35)	<0.001	−2.22 (−3.71, −0.73)	0.004	−2.13 (−3.73, 0.52)	0.010
Age (years)	–	–	−0.17 (−0.25, −0.09)	<0.001	−0.18 (−0.26, −0.09)	<0.001	–	–
Sex (male)	–	–	−0.10 (−1.65, 1.45)	0.897	−0.30 (−1.89, 1.28)	0.705	–	–
Years of education	–	–	1.61 (1.08, 2.13)	<0.001	1.37 (0.81, 1.94)	<0.001	–	–
Accommodation type								
Wendyhouse/backyard dwelling vs. shack	–	–	–	–	2.34 (0.55, 4.13)	0.010	–	–
Own/family house vs. shack	–	–	–	–	1.58 (−0.20, 3.36)	0.081	–	–
Depressive symptoms (CES-D > 16)	–	–	–	–	0.50 (−1.44, 2.44)	0.615	–	–
Principal Components								
*Childhood Psychosocial Variables* component	–	–	–	–	0.37 (−0.39, 1.13)	0.343	–	–
*Current Psychosocial Variables* component	–	–	–	–	0.14 (−0.52, 0.80)	0.677	–	–
*Experience of Childhood Trauma* component	–	–	–	–	−0.41 (−1.08, 0.26)	0.232	–	–
Propensity score	–	–	–	–	–	–	−1.72 (−2.44, −1.01)	<0.001

95% CI, 95% confidence interval; CES-D, Centre for Epidemiological Studies-Depression.

## Discussion

Our data show that multiple psychosocial factors are different in persons with HIV compared to persons without HIV, and are associated with lower scores on cognitive testing. These psychosocial factors account for a proportion of the difference in cognitive performance between persons with and without HIV. This confounding remains despite efforts to control for these factors by comparison with an appropriate control group and by controlling for standard variables typically measured in HIV-cognition studies (i.e., age, sex and years of education). This has important implications for the interpretation of cognitive data in HIV-cognition studies. Misinterpreting low cognitive test performance risks overestimation of cognitive impairment on a population level, which has the potential to raise anxiety amongst persons with HIV and increase stigma and discrimination towards them. False positive outcomes could burden services and waste limited healthcare resources and lead to misguided investigation in biomarker and treatment research.

Despite efforts to identify a sociodemographically similar control group of persons without HIV, there were differences in most psychosocial factors. Persons with HIV were more likely than persons without HIV to live in a shack, or grow up without basic assets such as running water or a flush toilet. They were more likely to be brought up by primary caregivers with lower education and occupational background, and attend lower quality schools than those without HIV. It is striking that most of these psychosocial variables, both current and in childhood, were negatively associated with cognitive scores as an adult. For example, the assets someone had growing up, or the level of education of their primary caregiver, corresponds to measurable differences in cognitive performance as an adult. Our PCA and multivariable analysis further underlines the importance of early life events in the testing of cognitive performance [16,26,27].

Psychosocial variables have been shown to impact cognitive performance in other studies, including in persons with HIV [3,5,28,29]. For example in the Women's Interagency HIV Study of 1521 women (1019 living with HIV), the effect sizes for HIV status on cognitive performance accounted for just 0.05 to 0.09 SD units, which was less than for years of education, age, race, income, and reading level [30]. The reasons for this are complex and reviewed extensively elsewhere [27]. It should be emphasized that these psychosocial variables correspond to lower performance on cognitive tests, rather than to cognitive impairment *per se*. Performance on cognitive tests is a weak proxy of actual cognitive function due to confounding of cultural, linguistic and background factors. This is reflected by the fact that it is typical for cognition studies (both within and outside the HIV field) to adjust for standard demographic variables to obtain more accurate estimates of cognitive performance [31]. Such an adjustment would not be necessary, and indeed would introduce bias, if these factors were considered to actually lower cognitive function and/or cause cognitive impairment. Of note, ethnicity is typically included in such adjustments and is specified for adjustment in the HAND criteria [13]. This is presumably a crude indicator of other social and environmental factors and we propose that studies avoid using race and ethnicity as proxies for social and environmental factors and, where possible, directly measure those variables instead. Race and ethnicity are social constructs whose meaning originates in and is dependent on social, political and historical forces, including as a variable in studies should be done thoughtfully and responsibility and is only justified where the aim is to understand and redress racial and ethnic inequity [32].

The level of evaluation of psychosocial factors undertaken in our study is not feasible in most HIV-cognition studies, and we are not advocating this detailed approach be widely adopted. To do so would be impractical and key variables may vary between settings and over time. Rather, we suggest that HIV-cognition studies should acknowledge this potential confounder and carefully consider the effect of HIV on cognitive performance [5,30,33]. Researchers should avoid making classifications of disease burden based solely or heavily on psychometric neuropsychological methods, that is, a reliance on the result of cognitive testing without clinical context. Ideally cognitive impairment from HIV-associated brain injury should only be diagnosed after a clinical assessment by a neurologist or clinical neuropsychologist.

Our data support the recent consensus statement from the International HIV-Cognition Working Group for a new approach to classification of cognitive impairment in persons with HIV, in particular that language be destigmatized such that those performing below a threshold on cognitive tests are referred to as having ‘low cognitive performance’ rather than being labelled with cognitive impairment, unless supported by additional evidence of clinical impact [34]. It is critical to accurately distinguish genuine cognitive impairment in a person with HIV from low cognitive performance due to psychosocial confounds, so that limited services can be targeted towards those that need it.

This study has limitations. The variables we chose to measure were based on our knowledge of the environment and may not have measured all the key variables in that population. For example, in a previous study from a similar area we identified food insecurity, a marker of extreme poverty, as the main parameter associated with cognitive performance [5]. We did not measure food insecurity in the current cohort. The generalizability of our findings to other settings is unclear; a different environment may have a different set of variables affecting cognitive performance, although we would argue that the principles remain relevant. The generalizability of our findings might also be limited by restrictions on our recruitment to exclude those with conditions that could contribute to cognitive problems, including hazardous alcohol use and substance use. These factors (particularly hazardous alcohol use) are common in South Africa, as illustrated by the screening exclusion rates reported elsewhere [17]. They have been shown to interact with HIV, and with psychosocial factors, to contribute to cognitive impairment, but were not examined in our cohort [35–37]. Similarly, those with vertical HIV acquisition were excluded to avoid confounding effects on cognitive outcomes related to neurodevelopmental challenges that can arise from early-life HIV exposure, and hence not represented in our cohort. All our participants with HIV were receiving efavirenz-based antiretroviral therapy, which is known to cause cognitive side effects [38]. Indeed we observed an improvement in cognitive performance following switch to dolutegravir-based antiretroviral therapy in the parent study [17]. This could have impacted cognitive scores and decreased their association with psychosocial variables. There were more depressive symptoms in those without HIV, possibly due to better access and engagement with healthcare in this group. Unexpectedly, the SECTV measure identified less violence experienced by persons with HIV and less childhood exposure to violence was associated with lower cognitive performance; while this could relate to urban versus rural living we did not have enough information to explore this in detail, and it highlights the complexity of lived experience in these settings. Our study measures took half a day, for which participants were compensated, which could have introduced selection bias towards those without full-time employment. Lastly, our measures for childhood were based on recall and may have been impacted by recall bias. We chose recall for 8–10 years as we felt this was the earliest they were likely to remember, but it may be that the key stages for cognitive development are earlier.

In conclusion, many psychosocial factors are associated with lower cognitive performance and differences in these between persons with and without HIV partially explains lower scores among persons with HIV. Given this effect, and the possibility of further unmeasured confounding, analyses of cognitive performance in persons with HIV should be interpreted with caution to avoid overestimation of cognitive impairment.

## Acknowledgements

We would like to acknowledge the study team, especially Zimkhitha Ndinga and Teboho Linda, and the research study participant volunteers.

Funding: Research reported in this publication was supported by the South African Medical Research Council, with funds received from the South African National Department of Health and the UK Medical Research Council, with funds received from the UK Government's Newton Fund.

### Conflicts of interest

There are no conflicts of interest.

## Supplementary Material

Supplemental Digital Content
