# Supplementary material for: Psychosocial factors account for a proportion of the difference in cognitive performance between persons with and without HIV
Source: AIDS. 2024 Dec 19;39(4):393–402. doi: 10.1097/QAD.0000000000004080 (PMC11872261; doi:10.1097/QAD.0000000000004080)
Supplement: Supplemental Digital Content [file aids-39-393-s001.docx]

**Supplementary materials**

**Methods**

**Cognitive domains, tests and outcomes**

The domains, tests, and outcome variables were: (1) *executive functioning,* Color Trails Test 2 – completion time (in seconds); Wisconsin Card Sorting Test – total score; (2) *verbal learning and memory,* Hopkins Verbal Learning Test-Revised – total across the three immediate recall trials, total on the delayed recall trial; (3) *visuospatial learning and memory,* Brief Visuospatial Memory Test-Revised – total across the immediate recall trials, total on the delayed recall trial; (4) *verbal fluency,* category fluency test – total number of animals / total number of fruits and vegetables named in 1 minute; (5) *attention/working memory,* Wechsler Adult Intelligence Scale-Third Edition (WAIS-III) Digit Span subtest – total raw score; (6) *processing speed* – completion time (in seconds); WAIS-III Digit Symbol Coding subtest – total raw score; WAIS-III Symbol Search – total raw score; (7) *motor skills,* Grooved Pegboard Test nondominant hand – completion time (in seconds); Finger Tapping Test nondominant hand – completion time (in seconds).

**Processing of cognitive data into z-scores and T-scores**

Raw scores were standardized to z-scores using data from the group of people without HIV (*N* = 95). For each cognitive outcome variable, the z-score was calculated by subtracting the persons without HIV group mean from the raw score, divided by the persons without HIV group standard deviation. z-scores were reversed if a higher score indicates a worse test performance (i.e., Color Trails Test 1 and 2, Grooved Pegboard Test and Finger Tapping Test). The z-scores were then converted to T-scores (M = 50, SD = 10). If participants had z-scores greater than 5 SD below the mean, the conversion to a T-score resulted in negative T-score. In these cases, we assigned a score of zero, the lowest possible T-score to maintain the clinical significance of such poor performance. Domain T-scores were calculated by taking the average of T-scores of the cognitive outcomes within each domain. Global T-score was calculated by taking the average across-domain T-scores. All statistical analyses were done using global T-scores.

**Principal component analysis**

Variables were scaled and reversed scored if a higher score indicated worse psychosocial circumstances (this was the case for the variables *occupation category, primary caregiver occupation category, CTQ score* and *SECTV score*). Missing values in the dataset were imputed using the Predictive Mean Matching method from the Multiple Imputation by Chained Equations (MICE) package in R. The Kaiser-Meyer-Olkin test measured the suitability of data for factor analysis. Cattell’s criteria (i.e., examination of the scree plot) was used to determine the number of components to retain. After the number of components was determine using the scree, we applied a varimax rotation. For an item to be retained in a component, it needed to have a factor loading > 0.4 and no higher loading on another factor. The PCA scores were then extracted for each component to use in further analyses.

Note that *accommodation* *type* and *depression* (based on the CES-D score) were not included in the PCA because they are categorical variables. Hence, rather than being included in the composite scores they were instead kept as individual variables in subsequent analyses. Similarly, the standard demographic variables (*age, sex, years of education)* and *HIV status* were also kept as separate individual variables in subsequent analyses.

**Propensity score modelling**

To conduct these analyses, we started by fitting a logistic regression model with HIV status as the outcome – predictors were any factors that might confound the association between HIV status and global cognitive performance (i.e., psychosocial variables as well as standard demographic variables). Using this model, the predicted value from that model for each individual (i.e., the expected probability of being HIV positive, given the person’s covariable values) was calculated and these scores were used as a single predictor variable, along with HIV status, in a model with global T-score as the outcome. This allowed us to adjust for variables significantly associated with HIV status, so we could determine the direct effect of HIV status on global T-score. The change in the regression co-efficient for HIV status was calculated by comparing to a separate simpler model, with HIV status as a single predictor of global T-score. This indicated the difference in global T-score when taking into account variables associated HIV status, compared to HIV status alone. The same was calculated with standard demographic variables (age, gender, level of education) also added to the model.

**Results**

**Principal component analysis**

The PCA revealed three components that cumulatively explained 57.5% of the total variance in the dataset (Supp table 1). The first (labelled as *Childhood Psychosocial Variables*) accounted for 29.7% of the variance, the second (*Current Psychosocial Variables*) accounted for an additional 15.2% of the variance, and the third (*Experience of Childhood Trauma*) accounted for an additional 12.6%. While SECTV score loaded primarily onto this third component, its secondary loading was on *Childhood Psychosocial Variables* (-0.41), indicating a potential overlap in the constructs measured by these components.

*Supp table 1.*

Results from PCA analysis demonstrating the three principal components along with the factors included and their loadings.

| Childhood Psychosocial Variables | | Current Psychosocial Variables | | Experience of Childhood Trauma | |
| --- | --- | --- | --- | --- | --- |
| Variables | Factor loading | Variables | Factor loading | Variables | Factor loading |
| Primary caregiver occupation category | 0.66 | Current asset index | 0.45 | CTQ score | 0.86 |
| Childhood asset index | 0.79 | Monthly income | 0.86 | SECTV score | 0.66 |
| Primary school quintile | 0.72 | Occupation category | 0.73 |  |  |
| Secondary school quintile | 0.71 |  |  |  |  |
| Primary caregiver level of education | 0.71 |  |  |  |  |

*Note*. CTQ = Childhood Trauma Questionnaire; SECTV = Survey of Exposure to Community Violence.

**Propensity score model**

*Supp table 2.*

Results from the logistic regression model with HIV status as the outcome used to determine propensity scores

| Variables | *Odds Ratio* | 95% *CI* | *p* |
| --- | --- | --- | --- |
|  |  |  |  |
| Age (years) | 1.00 | 0.97 – 1.04 | .889 |
| Sex (male) | 0.79 | 0.39 – 1.59 | .499 |
| Years of education | 0.81 | 0.62 – 1.05 | .113 |
| *Childhood Psychosocial Variables* component | 0.65 | 0.46 – 0.90 | **.011** |
| *Current Psychosocial Variables* component | 1.01 | 0.75 – 1.36 | .954 |
| *Experience of Childhood Trauma* component | 1.01 | 0.74 – 1.36 | .966 |
| Accommodation type |  |  |  |
| Wendy house/backyard dwelling vs. shack | 0.66 | 0.27 – 1.56 | .349 |
| Own/family house vs. shack | 0.26 | 0.11 – 0.58 | **.001** |
| Depressive symptoms (CES-D > 16) | 0.45 | 0.20 – 1.00 | .051 |

*Note*. 95% *CI* = 95% confidence interval
